# Assessment of Health-Related Quality of Life in Adult Spinal Muscular Atrophy Under Nusinersen Treatment—A Pilot Study

**DOI:** 10.3389/fneur.2021.812063

**Published:** 2022-01-24

**Authors:** Andreas Thimm, Svenja Brakemeier, Kathrin Kizina, Juan Munoz Rosales, Benjamin Stolte, Andreas Totzeck, Cornelius Deuschl, Christoph Kleinschnitz, Tim Hagenacker

**Affiliations:** ^1^Department of Neurology and Center for Translational Neuro- and Behavioral Sciences, University Hospital Essen, Essen, Germany; ^2^Institute for Diagnostic and Interventional Radiology and Neuroradiology, University Hospital Essen, Essen, Germany

**Keywords:** spinal muscular atrophy, nusinersen, Neuro-QoL, antisense oligonucleotide, HFMSE, RULM

## Abstract

5q-Spinal muscular atrophy (SMA) is a severely disabling inherited neuromuscular disease that progressively reduces the motor abilities of affected individuals. The approval of the antisense oligonucleotide nusinersen, which has been shown to improve motor function in adult SMA patients, changed the treatment landscape. However, little is known about its impact on patients' quality of life (QoL), and there is still a need for adequate patient-reported outcome measures. In this study, we used the short form of the Neuro-QoL (Quality of Life in Neurological Disorders) for upper/lower extremity function to prospectively assess the health-related QoL of 17 adult SMA patients prior to initiation of nusinersen treatment and 2, 6, 10, and 14 months afterwards. At baseline, Neuro-QoL scores strongly correlated with motor function scores (Hammersmith Functional Motor Scale Expanded, HFMSE; Revised Upper Limb Module, RULM), but QoL did not increase significantly during the 14-month treatment period despite significant motor improvement as measured by HFMSE. Our results underline the need for novel, disease-specific assessments of QoL in SMA.

## Introduction

5q-Spinal muscular atrophy (SMA) is an autosomal-recessive inherited motor neuron disease caused by homozygous deletions or mutations in the survival of the motor neuron 1 gene (SMN1) on chromosome 5 encoding the SMN protein (1, 2). The copy number of SMN2, the homologous copy of SMN1, is considered the most important phenotypic modifier, inversely correlating with disease severity (3–5). The degeneration of anterior horn cells due to SMN1 loss or mutation leads to progressive muscular atrophy, tetraparesis and ultimately respiratory failure (6). With an incidence of ~1:10,000 (7, 8) it is in its most severe forms a common genetic cause of early infant mortality, while less affected patients typically reach adulthood. Based on the achievement of motor milestones and the age at disease onset, SMA is classically categorized into four subtypes (SMA type 0–3). SMA type 0 applies to severely affected neonates with a life expectancy reduced to <1 month (9, 10), SMA type 1 patients never learn to sit independently (“non-sitters”), SMA type 2 patients learn to sit but not to walk (“sitters”), and SMA type 3 patients learn to walk independently (“walkers”) (11). However, this classical view of SMA subtypes has been challenged in the era of disease-modifying treatments.

For decades, treatment options were restricted to supportive care. In 2017, the approval of nusinersen for the treatment of SMA patients of all ages, subtypes, and disease stages by the US Food and Drug Administration (FDA) and the European Medicines Agency (EMA) changed the therapeutic landscape. Recently, the treatment spectrum was broadened by Onasemnogene abeparvovec, a non-replicating recombinant adenovirus-associated virus serotype 9 (AAV9) containing the wild-type SMN1 gene (12), and Risdiplam, an orally applicable small molecule modifying SMN2 gene splicing (13).

Nusinersen is an intrathecally administered antisense oligonucleotide that increases the production of SMN protein by modifying the expression of the SMN2 gene (14, 15). It has been shown to improve survival and motor function in children (16, 17), and recently published data also proved its efficacy in adult SMA patients in terms of motor function as assessed by the Hammersmith Functional Motor Scale Expanded (HFMSE) (18). However, there are only limited data available about its impact on the quality of life (QoL) of adult SMA patients, and the selection of appropriate QoL outcome measures is still a matter of debate (19), but it is highly relevant for treatment monitoring in clinical practice and trials as well as for regulatory authorities. Indeed, identification of novel biomarkers and outcome measures seems mandatory for optimizing treatment approaches and predicting treatment response (20).

In this study, we aimed to evaluate the QoL of adult SMA patients under nusinersen treatment using the short forms of the Neuro-QoL (Quality of Life in Neurological Disorders) (21) for lower extremity function and upper extremity function to address the questions of whether nusinersen treatment improves health-related QoL in adult SMA patients, whether changes in QoL and motor function under treatment correlate with each other, and to what extent the Neuro-QoL questionnaire might be useful in this patient cohort.

## Methods

### Study Design and Participants

All examinations and treatments were carried out at the Department of Neurology of the University Hospital Essen from January 2019 until May 2021. Seventeen consecutive patients with genetically confirmed SMA type 2 or 3 treated with nusinersen were prospectively included. Health-related QoL was assessed using the short forms of the Neuro-QoL for upper and lower extremity function prior to treatment initiation, as well as 2, 6, 10 (17 patients), and 14 (15 patients) months afterwards. We concentrated on these subsets of the Neuro-QoL instead of including more domains to specifically evaluate the impact of nusinersen on motor function-related QoL and to avoid exhaustion of participants and interference with results of several other examinations as part of clinical routine. Motor function was evaluated concomitantly by means of the HFMSE and the Revised Upper Limb Module (RULM) score. Intrathecal nusinersen treatment was performed according to the official prescribing information for at least 14 months.

### Questionnaire and Motor Function Tests

The short forms of the Neuro-QoL for upper and lower extremity function are patient-reported outcome measures comprising 8 items, each referring to the activities of daily living requiring a certain motor function. Each question is answered on a 5-point rating scale (1–5), with higher scores (maximum 40) indicating a higher QoL. The HFMSE and RULM are well-established rating scales for motor function in SMA patients. The HFMSE consists of 33 items representing upper and lower extremity motor function, each scored on a 3-point scale (0–2) with higher scores (maximum 66) indicating greater motor function, while the RULM score represents the motor abilities of the upper extremities only with 18 items scored on a 3-point rating scale (0–2) and 1 item on a 2-point rating scale (0–1) (maximum 37).

### Statistical Analysis

Statistical analyses were performed using GraphPad Prism (version 9.1.0 for Windows, GraphPad Software, San Diego, California, USA). All data are presented as the mean, standard error of the mean, and *P*-values. Analyses were based on pre-post comparisons from baseline to months 2, 6, 10, and 14 using the Friedman test and Dunn's test as *post hoc* test. The *P*-values < 0.05 were considered to be statistically significant. Correlations were calculated using Spearman's rank correlation coefficient.

## Results

Seventeen patients with genetically confirmed SMA type 2 (23.5%) or type 3 (76.5%) were included in the study (11 male, 6 female, mean age 35.82 ± 13.94 years). For detailed demographic characteristics see Table 1 and Supplementary Table 1.

**Table 1:** Baseline characteristics and demographics of the included SMA patients.

Age (mean ± SEM, years)	35.82 ± 13.94
Sex	11 male, 6 female
SMA type	4 (23.5%) SMA type 2
	13 (76.5%) SMA type 3
Ambulatory	7 (41.2%)
Baseline HFMSE score (mean ± SEM)	23.88 ± 5.77
Baseline RULM score (mean ± SEM)	22.18 ± 3.44
Baseline Neuro-QOL score (mean ± SEM) for upper extremity function	28.06 ± 2.48
Baseline Neuro-QOL score (mean ± SEM) for lower extremity function	16.00 ± 2.28

**Figure 1:**
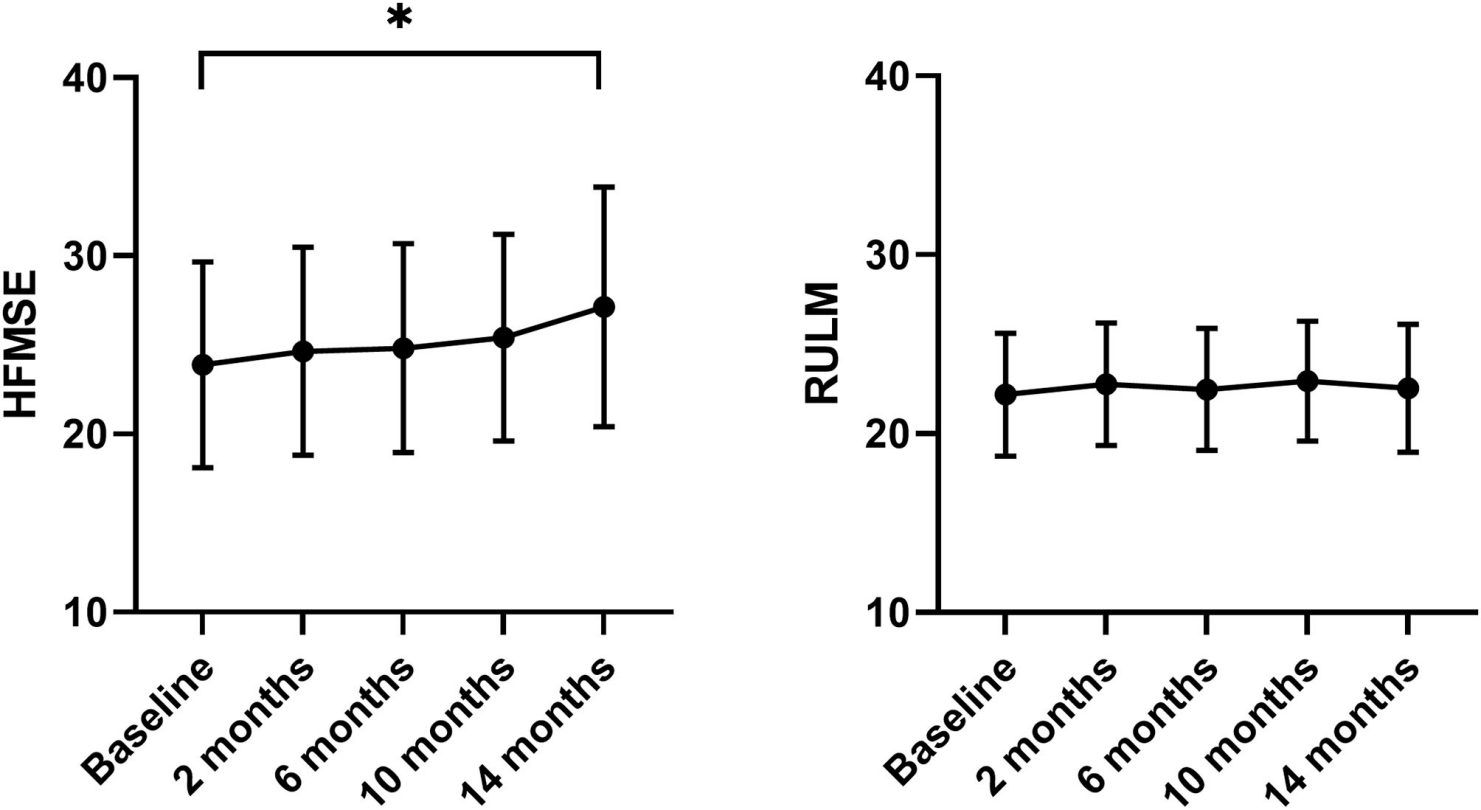
Motor function under nusinersen treatment. Mean HFMSE (left panel) and mean RULM scores (right panel) prior to (baseline) and 2–14 months after initiation of nusinersen treatment. MEAN ± SEM, *P < 0.05.

**Figure 2:**
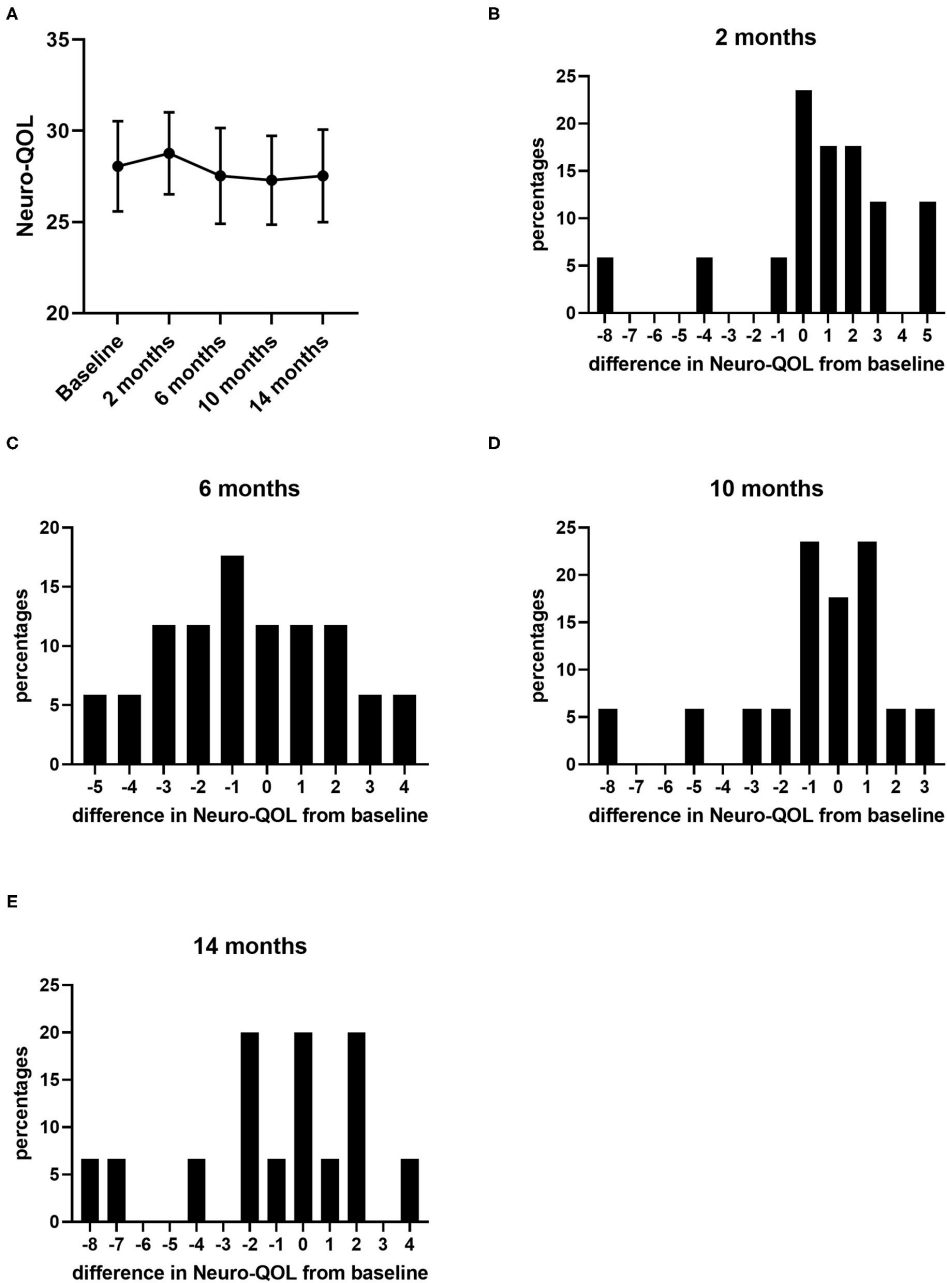
Quality of life measured by Neuro-QoL for upper extremity function under nusinersen treatment. Mean Neuro-QoL for upper extremity function (± SEM) during the 14-month treatment period **(A)** and distribution of differences in Neuro-QoL for upper extremity function from baseline 2 **(B)**, 6 **(C)**, 10 **(D)**, and 14 months **(E)** after treatment initiation. Each bar represents the percentage of patients who had improved/deteriorated to this extent.

**Figure 3:**
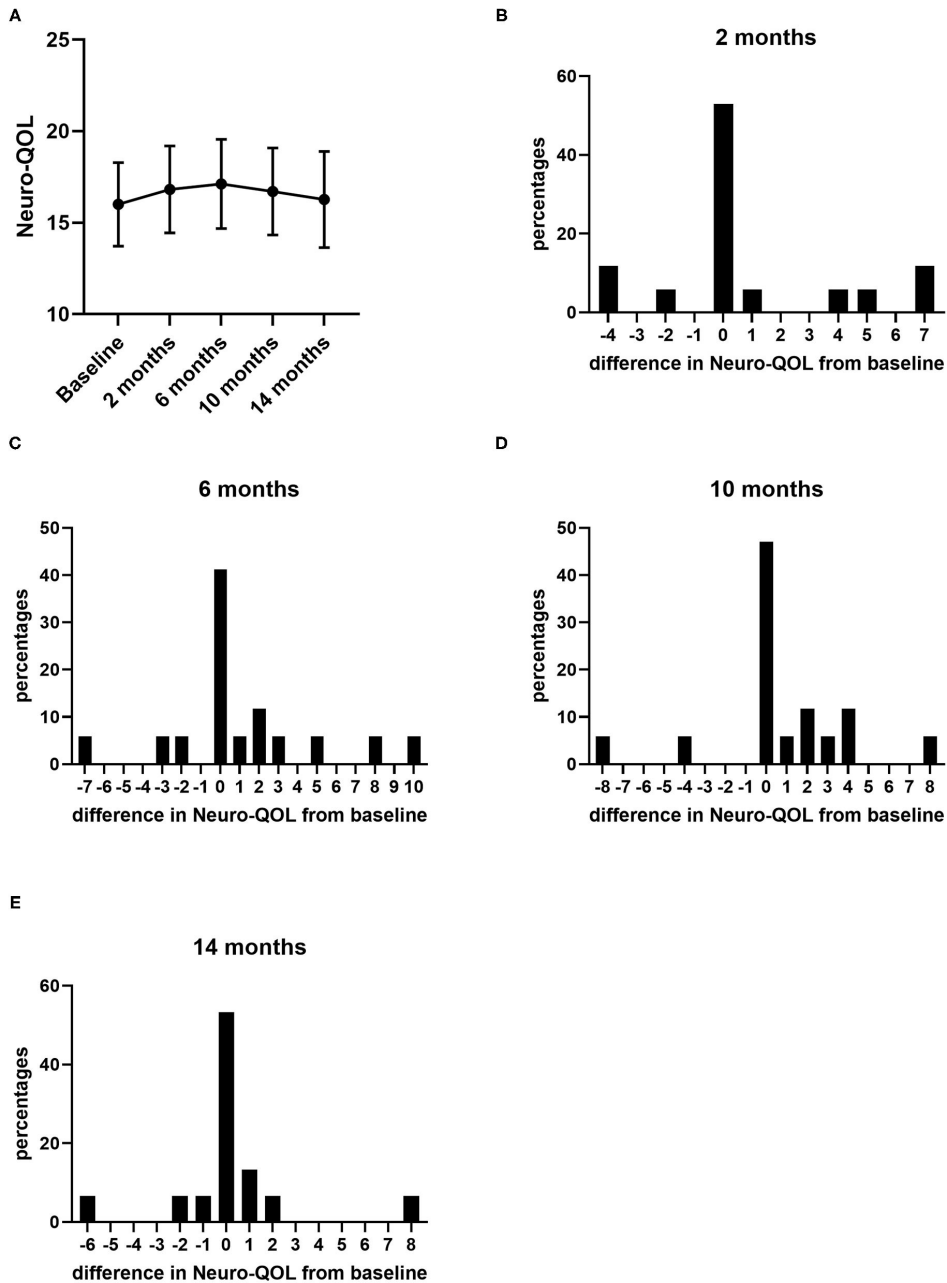
Quality of life measured by Neuro-QoL for lower extremity function under nusinersen treatment. Mean Neuro-QoL for lower extremity function (+SEM) during the 14-month treatment period **(A)** and distribution of differences in Neuro-QoL for lower extremity function from baseline 2 **(B)**, 6 **(C)**, 10 **(D)**, and 14 months **(E)** after treatment initiation. Each bar represents the percentage of patients who had improved/deteriorated to this extent.

During the 14 months of nusinersen treatment, the mean HFMSE scores increased (Figure 1; Table 2), which was significant compared to baseline at 14 months (+2.867 ± 0.867, *p* = 0.0488). On the other hand, RULM scores as a measure of upper extremity motor function did not show any significant change under treatment (Figure 1; Table 2). Correspondingly, the mean quality of life measured by the Neuro-QoL for the upper extremities did not improve during the 14-month period (Figure 2; Table 3). Changes compared to baseline at 2, 6, 10, and 14 months were not significant. After 14 months of treatment, 66.6% of patients (10 out of 15) had not improved on the Neuro-QoL for upper extremity function. In terms of the Neuro-QoL scores for lower extremity function, there was no significant change (Figure 3; Table 3) either. The highest results were reported at 6 months after treatment initiation (mean difference from baseline 1.12 ± 0.95, *p* = 0.415). Approximately 40% (7 out of 17) of patients showed an increase of at least 1 point on the Neuro-QoL for lower extremity function at this time.

**Table 2:** Changes in motor function scores vs. baseline.

	**HFMSE**	**RULM**
**2 months**		
Mean score (SEM)	24.65 (5.84)	22.87 (3.61)
Mean difference vs. baseline (SEM)	0.76 (0.42)	0.67 (0.90)
*P*-value	>0.999	>0.999
**6 months**		
Mean score (SEM)	24.82 (5.87)	22.47 (3.62)
Mean difference vs. baseline (SEM)	0.94 (0.57)	0.27 (0.47)
*P*-value	>0.999	>0.999
**10 months**		
Mean score (SEM)	25.41 (5.81)	22.93 (3.59)
Mean difference vs. baseline (SEM)	1.53 (0.84)	0.73 (0.67)
*P*-value	0.516	>0.999
**14 months**		
Mean score (SEM)	27.13 (6.72)	22.53 (3.57)
Mean difference vs. baseline (SEM)	2.87 (0.87)	0.33 (0.59)
*P*-value	0.049	>0.999

**Table 3:** Changes in health-related QOL vs. baseline.

	**Neuro-QOL, upper extremities**	**Neuro-QOL, lower extremities**
**2 months**		
Mean score (SEM)	28.76 (2.25)	16.82 (2.37)
Mean difference vs. baseline (SEM)	0.71 (0.76)	0.82 (0.78)
*P*-value	0.516	>0.999
**6 months**		
Mean score (SEM)	27.53 (2.63)	17.12 (2.44)
Mean difference vs. baseline (SEM)	−0.53 (0.61)	1.12 (0.95)
*P*-value	>0.999	0.415
**10 months**		
Mean score (SEM)	27.29 (2.44)	16.71 (2.37)
Mean difference vs. baseline (SEM)	−0.76 (0.64)	0.71 (0.83)
*P*-value	>0.999	>0.999
**14 months**		
Mean score (SEM)	27.53 (2.54)	16.27 (2.62)
Mean difference vs. baseline (SEM)	−1.00 (0.86)	0.20 (0.73)
*P*-value	>0.999	>0.999

**Figure 4:**
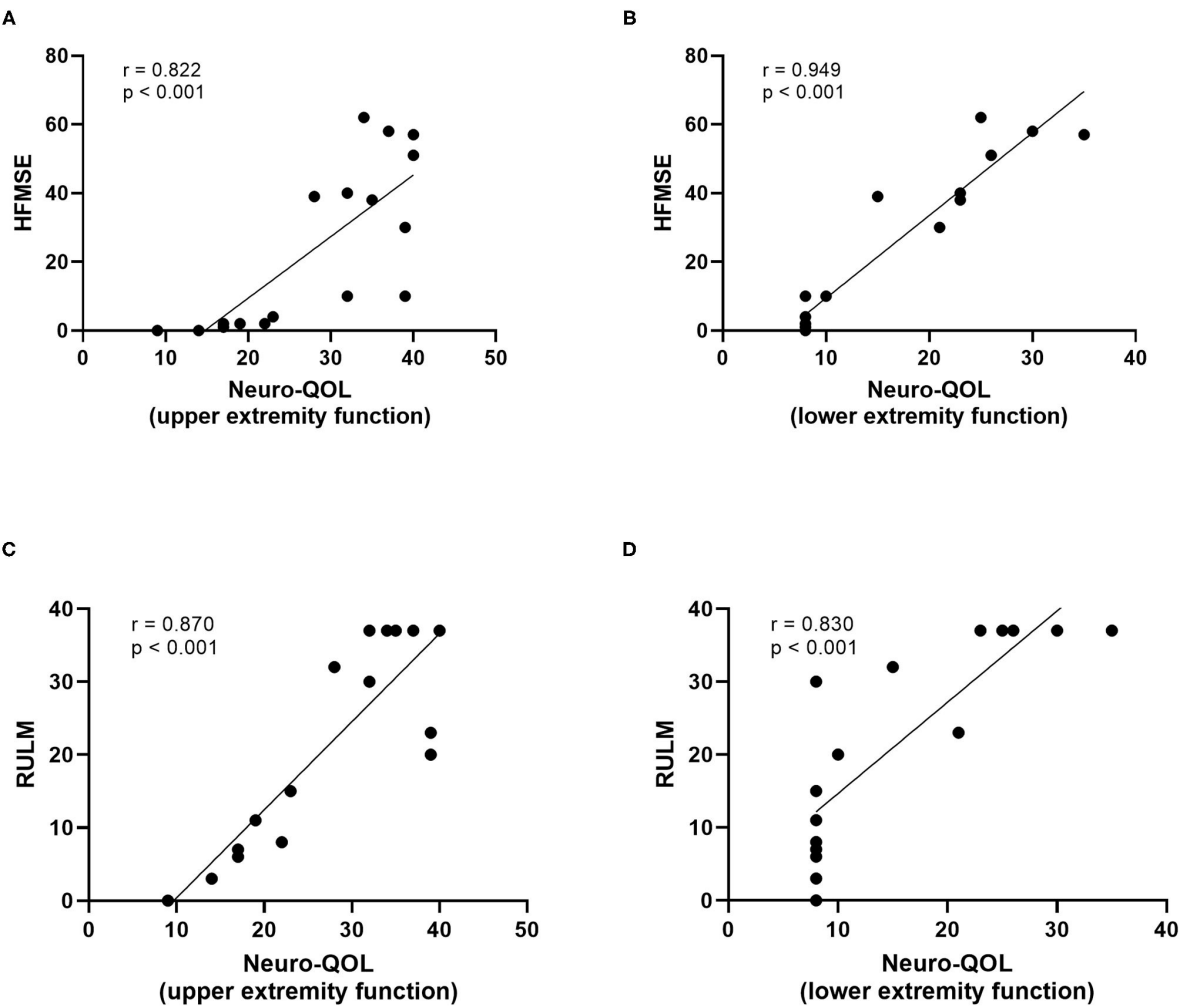
Correlatio of motor function scores and Neuro-QoL for upper/lower extremity function at baseline. HFMSE score vs. Neuro-QoL for upper extremity function **(A)**, HFMSE score vs. Neuro-QoL for lower extremity function **(B)**, RULM score vs. Neuro-QoL for upper extremity function **(C)**, RULM score vs. Neuro-QoL for lower extremity function **(D)**.

**Figure 5:**
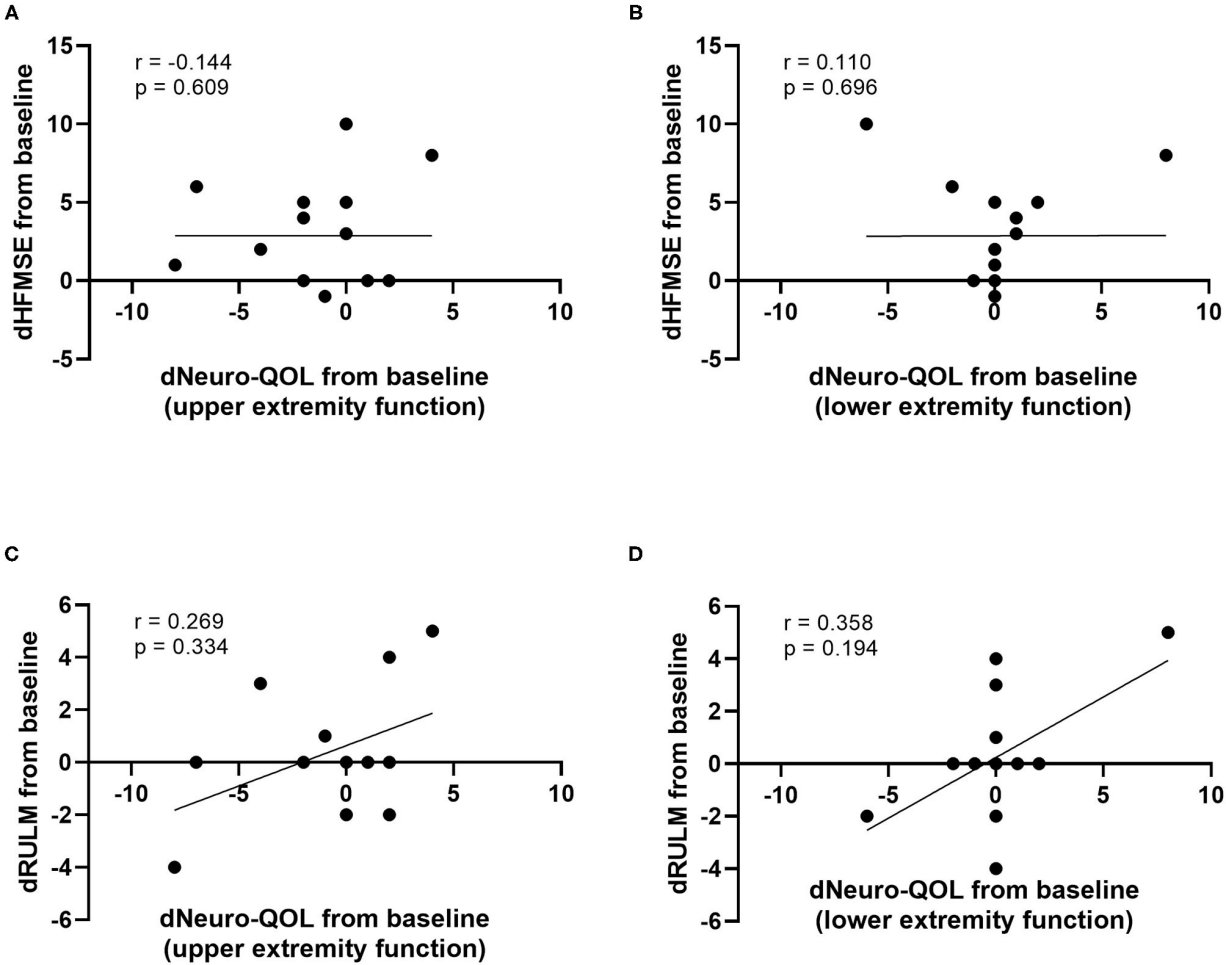
Correlation of differences in motor function scores compared to baseline (dHFMSE, dRULM) and differences in Neuro-QoL compared to baseline (dNeuro-QoL) after 14 months of treatment. dHFMSE vs. dNeuro-QoL for upper extremity function **(A)**, dHFMSE vs. dNeuro-QoL for lower extremity function **(B)**, dRULM vs. dNeuro-QoL for upper extremity function **(C)**, and dRULM vs. dNeuro-QoL for lower extremity function **(D)**.

At baseline, a strong correlation between motor function scores (HFMSE/RULM) and Neuro-QOL for upper and lower extremity function was evident (Figure 4). However, the increase in HFMSE score as an indicator of motor improvement under nusinersen treatment did not correlate with the observed changes in Neuro-QoL during the treatment period (Figure 5).

A subgroup analysis revealed differences between ambulatory (*n* = 5) and non-ambulatory (*n* = 11) patients. The included ambulatory SMA patients showed a significant increase in HFMSE scores at 14 months of treatment (+4.167 ± 1.078, *p* = 0.025), while the increase in non-ambulatory patients was highest at 10 months, but not significant (+2.333 ± 1.280, *p* = 0.177). RULM scores did not change significantly in both groups. Furthermore, both groups did not improve in Neuro-QoL scores over time.

## Discussion

Our study did not show an improvement in QoL measured by the mentioned subsets of the Neuro-QoL under nusinersen treatment, although HFMSE scores revealed a significant treatment response regarding motor function.

Several prior studies have shown impaired health-related QoL in SMA patients due to limitations in physical functioning, although their QoL in the mental domains seems to be comparable to that in healthy populations (22, 23). The available data suggest that during the natural disease course, the deterioration of motor function does not correlate with the decrease in QoL as measured by the PedsQL (Pediatric Quality of Life Inventory) (24) and the SF-36 (Short Form 36 Health Survey) (25), which is similar to the findings for Duchenne muscular dystrophy (DMD), another severely disabling hereditary neuromuscular disease with lower self-reported health-related QoL than in the general population (26). In DMD, QoL does not necessarily decline with decreasing physical abilities or increasing age (27, 28). Treatment with ataluren, a small molecule delaying motor function loss in patients with a non-sense mutation in DMD, is not associated with a significant increase in parent-reported QoL (29). Similarly, our data do not demonstrate an increase in health-related QoL measured by the Neuro-QoL questionnaires for upper/lower extremity function in adult SMA patients in the first year of nusinersen treatment despite improvements in motor function as indicated by increasing HFMSE scores. While prior to treatment initiation self-assessed QoL with regard to motor abilities was closely related to HFMSE ratings by examiners, the following changes in HFMSE scores under treatment were not reflected in the patients' own Neuro-QoL reports. This discrepancy raises several questions: Does nusinersen treatment not affect the QoL of SMA patients at all, although there might be an improvement in HFMSE scores regarded as clinically meaningful (30), or do the Neuro-QoL questionnaires miss the relevant effects for some reason?

Reviewing the literature, data on QoL in SMA patients under nusinersen treatment are scarce. A recent study investigated QoL in adult and pediatric SMA patients during the first 6 months of nusinersen treatment using different questionnaires (31). Mix et al. reported no significant difference in global QoL and depressiveness as evaluated with the Anamnestic Comparative Self-Assessment (ACSA) between adult SMA patients and healthy controls at study entry, while SMA patients showed reduced health-related QoL measured by the SF-36 in the dimensions “physical function” and “general health.” Interestingly, in this study, global QoL significantly decreased within the SMA group under treatment with nusinersen, while depressiveness remained stable, and only the scores in the “general health” dimension of the SF-36 increased significantly. The latter did not correlate with physical function represented by HFMSE scores, but Mix et al. demonstrated a weak negative correlation between HFMSE scores and global QoL and depressiveness. Unlike our study, no significant improvement in HFMSE scores was evident in this patient cohort, which might be due to the higher number of severely impaired non-ambulatory and SMA type 2 patients and the shorter observation period, as the HFMSE scores also increased significantly only after 14 months of treatment in our study. Furthermore, another study failed to demonstrate significant differences between patients under nusinersen treatment and patients with supportive care only based on proxy-reported QoL measures (32).

Although these data together with our own results allow for doubts regarding the efficacy of nusinersen treatment on QoL in adult SMA patients, they are contrary to recent studies reporting high satisfaction of patients under nusinersen treatment, especially regarding its effectiveness (33, 34), and at least transiently reduced fatigue (35). Moreover, treatment adherence is generally high in this patient cohort despite the obvious burden of intrathecal treatment. In light of these findings, it is questionable as to whether the Neuro-QoL is suitable for QoL assessment in SMA patients, while other disease-specific outcome measures have to be implemented. Although the Neuro-QoL questionnaires (Supplementary Material) address issues of autonomy relevant to the patients' daily life such as washing oneself, getting in and out of a car, or picking up coins from a table top, they are presumably insufficiently graded to capture relevant improvements in SMA patients. Besides, they do not represent motor functions not related to the extremities like swallowing which are nevertheless important for the patients' QoL. Recently, the Spinal Muscular Atrophy Health Index (SMA-HI) has been proposed as a novel means to capture the disease burden from a patient's perspective (36). Apart from that, treatment expectations have been shown to be even higher in less severely affected individuals (37), and other individual psychological factors (38) might have influenced the patient-reported QoL in our study. In fact, a depression episode was not evident in any of the included patients during the study. The small number of patients included might be another limitation of our study, but the cohort was rather homogenous, comprising predominantly non-ambulatory SMA type 3 patients, and the sample size sufficed to detect significant motor improvement. Furthermore, an observation period of more than 1 year after the beginning of nusinersen treatment should be sufficient for the detection of treatment effects.

In conclusion, nusinersen treatment does not lead to an increase in health-related QoL of adult SMA patients as measured by the Neuro-QoL for upper/lower extremity function. Disease-specific questionnaires should be used to re-evaluate the impact of nusinersen treatment on QoL in future studies.

## Data Availability Statement

The raw data supporting the conclusions of this article will be made available by the authors, without undue reservation.

## Ethics Statement

The studies involving human participants were reviewed and approved by Ethics Committee of the University Duisburg-Essen (approval number 18–8285-BO), Robert-Koch-Strasse 9-11, 45147 Essen, Germany. The patients/participants provided their written informed consent to participate in this study.

## Author Contributions

ATh, TH, and CK: conceptualization. ATh and TH: methodology and formal analysis. ATh, SB, KK, JM, BS, ATo, CD, and TH: investigation. ATh, SB, KK, JM, BS, ATo, and TH: resources. ATh: writing – original draft. ATh, SB, KK, JM, BS, ATo, CD, CK, and TH: writing – review and editing. All authors contributed to the article and approved the submitted version.

## Funding

This work was supported by the Universitätsmedizin Essen Clinician Scientist Academy (UMEA) and sponsored by Biogen (Cambridge, MA, USA). The funder was not involved in the study design, collection, analysis, interpretation of data, the writing of this article or the decision to submit it for publication.

## Conflict of Interest

KK and BS received travel reimbursement and speaker honoraria from Biogen. CK received honoraria from Biogen and Roche. TH received honoraria from Novartis, Biogen, and Roche and research support from Biogen, Roche, and AveXis. The remaining authors declare that the research was conducted in the absence of any commercial or financial relationships that could be construed as a potential conflict of interest.

## Publisher's Note

All claims expressed in this article are solely those of the authors and do not necessarily represent those of their affiliated organizations, or those of the publisher, the editors and the reviewers. Any product that may be evaluated in this article, or claim that may be made by its manufacturer, is not guaranteed or endorsed by the publisher.
