# Supplementary material for: Assessment of Health-Related Quality of Life in Adult Spinal Muscular Atrophy Under Nusinersen Treatment—A Pilot Study
Source: Front Neurol. 2022 Jan 24;12:812063. doi: 10.3389/fneur.2021.812063 (PMC8818760; doi:10.3389/fneur.2021.812063)
Supplement: Supplementary file 1 [file Table_1.DOCX]

**Table 1. Demographic characteristics of included SMA patients in detail**

| **patient** | **gender** | **age** (years) | **age at symptom onset** | **SMA type** | **SMN2 copy number** | **current ambulatory status** |
| --- | --- | --- | --- | --- | --- | --- |
| P1 | female | 24 | 11 months | 2 | 3 | sitter |
| P2 | male | 53 | 15 years | 3 | 7 | walker |
| P3 | female | 34 | 2 years | 3 | 3 | sitter |
| P4 | male | 57 | 2 years | 3 | 4 | Sitter |
| P5 | male | 28 | 6 months | 2 | 3 | sitter |
| P6 | male | 31 | 6 years | 3 | 4 | walker |
| P7 | male | 73 | 5 years | 3 | 4 | Sitter |
| P8 | female | 46 | 15 years | 3 | 4 | Walker |
| P9 | male | 22 | 6 years | 3 | 4 | Sitter |
| P10 | female | 25 | 4 months | 2 | 3 | Sitter |
| P11 | female | 27 | 12 years | 3 | 4 | walker |
| P12 | female | 40 | 2 years | 3 | 2 | sitter |
| P13 | male | 39 | 16 years | 3 | 4 | walker |
| P14 | male | 42 | 2 years | 3 | 3 | Sitter |
| P15 | male | 28 | 7 years | 3 | 3 | Walker |
| P16 | male | 28 | 5 years | 3 | 4 | Walker |
| P17 | male | 22 | 7 months | 2 | 3 | sitter |
