# Supplementary material for: Assessment of Health-Related Quality of Life in Adult Spinal Muscular Atrophy Under Nusinersen Treatment—A Pilot Study
Source: Front Neurol. 2022 Jan 24;12:812063. doi: 10.3389/fneur.2021.812063 (PMC8818760; doi:10.3389/fneur.2021.812063)
Supplement: Supplementary file 2 [file Data_Sheet_1.PDF]

Lower Extremity Function (Mobility) – Short Form

Please respond to each question or statement by marking one box per row.

		Without any difficulty	With a little difficulty	With some difficulty	With much difficulty	Unable to do
PFC45	Are you able to get on and off the toilet?...	☐ 5	☐ 4	☐ 3	☐ 2	☐ 1
PFA30	Are you able to step up and down curbs?...	☐ 5	☐ 4	☐ 3	☐ 2	☐ 1
PFA56	Are you able to get in and out of a car?	☐ 5	☐ 4	☐ 3	☐ 2	☐ 1
PFA45	Are you able to get out of bed into a chair?	☐ 5	☐ 4	☐ 3	☐ 2	☐ 1
PFA12	Are you able to push open a heavy door? ..	☐ 5	☐ 4	☐ 3	☐ 2	☐ 1
PFA53	Are you able to run errands and shop?.......	☐ 5	☐ 4	☐ 3	☐ 2	☐ 1
PFA31	Are you able to get up off the floor from lying on your back without help?...............	☐ 5	☐ 4	☐ 3	☐ 2	☐ 1
PFA23	Are you able to go for a walk of at least 15 minutes?	☐ 5	☐ 4	☐ 3	☐ 2	☐ 1

©2008-2018 David Cella and the PROMIS Health Organization on behalf of the National Institute for Neurological Disorders and Stroke (NINDS). Used with permission.
