# Supplementary material for: Assessment of Health-Related Quality of Life in Adult Spinal Muscular Atrophy Under Nusinersen Treatment—A Pilot Study
Source: Front Neurol. 2022 Jan 24;12:812063. doi: 10.3389/fneur.2021.812063 (PMC8818760; doi:10.3389/fneur.2021.812063)
Supplement: Supplementary file 3 [file Data_Sheet_2.PDF]

Upper Extremity Function (Fine Motor, ADL) – Short Form

Please respond to each question or statement by marking one box per row.

		Without any difficulty	With a little difficulty	With some difficulty	With much difficulty	Unable to do
PFA40	Are you able to turn a key in a lock?..........	☐ 5	☐ 4	☐ 3	☐ 2	☐ 1
PFA50	Are you able to brush your teeth?	☐ 5	☐ 4	☐ 3	☐ 2	☐ 1
NQUEX44	Are you able to make a phone call using a touch tone key-pad?	☐ 5	☐ 4	☐ 3	☐ 2	☐ 1
PFB21	Are you able to pick up coins from a table top?...	☐ 5	☐ 4	☐ 3	☐ 2	☐ 1
PFA43	Are you able to write with a pen or pencil?	☐ 5	☐ 4	☐ 3	☐ 2	☐ 1
PFA35	Are you able to open and close a zipper?...	☐ 5	☐ 4	☐ 3	☐ 2	☐ 1
PFA55	Are you able to wash and dry your body?.	☐ 5	☐ 4	☐ 3	☐ 2	☐ 1
PFB26	Are you able to shampoo your hair?	☐ 5	☐ 4	☐ 3	☐ 2	☐ 1

©2008-2018 David Cella and the PROMIS Health Organization on behalf of the National Institute for Neurological Disorders and Stroke (NINDS). Used with permission.
